# SARS-CoV-2: lessons from both the history of medicine and from the biological behavior of other well-known viruses

**DOI:** 10.2217/fmb-2021-0064

**Published:** 2021-09-01

**Authors:** Sirio Fiorino, Fabio Tateo, Dario De Biase, Claudio G Gallo, Paolo E Orlandi, Ivan Corazza, Roberta Budriesi, Matteo Micucci, Michela Visani, Elisabetta Loggi, Wandong Hong, Roberta Pica, Federico Lari, Maddalena Zippi

**Affiliations:** ^1^Internal Medicine Unit, Budrio Hospital, Budrio (Bologna), Azienda USL, Bologna, 40054, Italy; ^2^Institute of Geosciences & Earth Resources, CNR, c/o Department of Geosciences, Padova University, 35127, Italy; ^3^Department of Pharmacy & Biotechnology, University of Bologna, Bologna, 40126, Italy; ^4^Fisiolaserterapico Emiliano, Castel San Pietro Terme, Bologna, 40024, Italy; ^5^Unit of Radiology, Maggiore Hospital, Bologna, 40133, Italy; ^6^Department of Experimental, Diagnostic & Specialty Medicine, University of Bologna, Bologna, 40126, Italy; ^7^Department of Pharmacy & Biotechnology, Alma Mater Studiorum-University of Bologna, Bologna, 40126, Italy; ^8^Hepatology Unit, Department of Medical & Surgical Sciences, University of Bologna, Bologna, 40126, Italy; ^9^Department of Gastroenterology & Hepatology, First Affiliated Hospital of Wenzhou Medical University, Wenzhou City, Zhejiang, 325035, PR China; ^10^Unit of Gastroenterology & Digestive Endoscopy, Sandro Pertini Hospital, Rome, 00157, Italy

**Keywords:** COVID-19, infectious diseases, pathogenesis, SARS-CoV-2 pandemic, treatment

## Abstract

SARS-CoV-2 is the etiological agent of the current pandemic worldwide and its associated disease COVID-19. In this review, we have analyzed SARS-CoV-2 characteristics and those ones of other well-known RNA viruses viz. HIV, HCV and Influenza viruses, collecting their historical data, clinical manifestations and pathogenetic mechanisms. The aim of the work is obtaining useful insights and lessons for a better understanding of SARS-CoV-2. These pathogens present a distinct mode of transmission, as SARS-CoV-2 and Influenza viruses are airborne, whereas HIV and HCV are bloodborne. However, these viruses exhibit some potential similar clinical manifestations and pathogenetic mechanisms and their understanding may contribute to establishing preventive measures and new therapies against SARS-CoV-2.

At the end of 2019 an epidemic, caused by a novel ssRNA human-infecting coronavirus, referred to as SARS-CoV-2, broke out in Wuhan (China) and subsequently spread worldwide. Its related disease, COVID-19, is characterized both by different degrees of severity and by a wide spectrum of signs and symptoms, including cough, sore throat, fever, shortness of breath, sudden onset of anosmia, ageusia or dysgeusia, nausea, vomiting and diarrhea [1]. This epidemic was declared a ‘public health emergency of international concern’ by the International Health Regulations Emergency Committee of the World Health Organization [2], and to date has caused more than 3.8 million deaths around the world (WHO, Coronavirus Disease COVID-19 Dashboard, accessed on 19/06/2021). Despite the strong efforts of researchers, currently only some drugs potentially active against this virus have been introduced in the clinical practice, as well as some vaccines have been urgently approved [3,4]. New diagnostic/therapeutic strategies must consider a wide spectrum of factors, concerning not only the viral characteristics, including the epidemiology, the structure, the route of infection and the pathogenetic mechanisms of SARS-CoV-2, but also the host’s features, such as age, co-morbidities, the reactivity of the immune system (IS) and the type of immune response (IR), and several critical physical parameters (climate, temperature, humidity and the duration of sunshine of the geographical regions where the pandemics spread). Accordingly, we have analyzed the features of some RNA viruses, widely occurred in mankind worldwide for several decades and responsible for a high burden of morbidity and mortality, with the purpose to find out possible analogies in the structure, the mechanisms of pathogenicity and the clinical course among these pathogens. In particular, we have decided to consider the following RNA viruses in our paper: HIV, HCV and Influenza viruses (IVs). HCV and HIV infect some classes of immune cells (mainly T cells and macrophages) using them as carriers via blood circulation. Two very important points have to be considered: the epidemiological factors and the mechanisms involved in the ability of IVs, HCV and HIV in inducing infectious diseases (IDs) in mankind. These viruses have been studied longer than SARS-CoV-2 and a better comprehension, concerning their biological behavior in human pathology, has been reached. We have considered the data available in the literature, in order to get useful lessons from the analysis of SARS-CoV-2 and the above-mentioned RNA viruses. This approach may contribute to allow us to exploit the historical knowledge, mainly with regard to the long-term effects of viral infections, which caused pandemics in the past. This strategy may also improve the understanding of the biological behavior of SARS-CoV-2, by identifying possible common viral characteristics and pathogenic mechanisms of the pathogens described above [5].

## Biology & pathogenesis of SARS-CoV-2, HIV, HCV & IVs in mankind SARS-CoV-2 structure & pathogenesis

**Figure 1.**
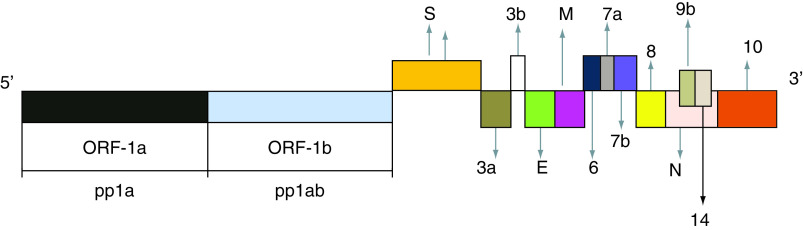
SARS-CoV-2 genome consists of a positive 5’-capped and 3’-polyadenylated ssRNA. The viral genome sequence is approximately 30,000 bases in length. OFR1a and OFR1b genes are located on the 5’-end and codify two long polypeptides, defined as pp1a and pp1ab, respectively. These polypeptides are cleaved into 16 Nsps (not shown), from Nsps 1 to Nsps 16, such as some transmembrane domains, including Nsp4 and Nsp6 (see text) as well as Nsp12 Pol/RdRp. This enzyme is needed for efficient replication and transcription of the viral RNA genome. Furthermore, SARS-CoV-2 genome on 3’-terminus encodes four structural proteins, the nucleocapsid **(N)** protein, the matrix **(M)** protein, the small envelope **(E)** protein and the spike **(S)** glycoprotein and also some accessory proteins, like ORF 3a, 3 b, 6, 7a, 7b, 8, 9b, 10 and 14. Nsp: Nonstructural protein; OFR: Open-reading frame; Pol/RdRp: RNA-dependent RNA polymerase.

SARS-CoV-2 is an enveloped nonsegmented virus with a spherical-shape [6]. The envelope is composed of a lipid bilayer, with spike proteins, emerging from the surface of the viral particles. Each virion consists of a positive 5’-capped and 3’-polyadenylated ssRNA. The viral genome sequence is approximately 30,000 bases in length (Figure 1), including 14 open-reading frames (ORFs) [7]. *OFR1a* and *OFR1b* genes are located on the 5’-end and codify two long polypeptides, defined as pp1a and pp1ab, respectively. They are cleaved into 16 nonstructural proteins (Nsps). To date, the identified activities of these molecules are the following: Nsp1 (modulation of viral RNA replication and inhibition of protein translation within the infected cells); Nsp2 (regulation of cell-signaling cascades, controlling the survival of infected cells); Nsp3, a protease cleaving the translated polyprotein into its different components; Nsp4, a protein including the transmembrane domain 2, probably anchoring a structure involved in viral transcription and replication to endoplasmic reticulum (ER) membranes; Nsp5, a proteinase, contributing to viral replication via polyprotein processing; Nsp6, a transmembrane domain, exerting a role in the early activation of autophagosomes from ER of infected cells; Nsp7, a cofactor with a hexadecameric structure, forming a complex with nsp8 and nsp12, modulating viral replication; Nsp8, a cofactor with a hexadecameric structure, forming a-complex with nsp7, regulating viral replication; Nsp9, a viral protein binding to single-stranded SARS-CoV-2 RNA, involved in viral replication by functioning as an ssRNA-binding protein; Nsp10, a growth factor-like protein, including two zinc-binding motifs. It stimulates both nsp14 3’–5’ exoribonuclease and nsp16 2’-*O*-methyltransferase activities, during viral transcription and with an essential role in viral mRNAs cap methylation; Nsp11, unknown activity; Nsp 12, a RNA-dependent RNA polymerase (RdRp), requested for efficient replication and transcription of the viral genome; Nsp13, a RNA 5’-triphosphatase includes a Zinc-binding domain, regulating replication and transcription and an NTPase/helicase domain, which binds ATP; Nsp14, including a proofreading Exoribonuclease domain-ExoN/nsp14 with an Exoribonuclease activity, functioning in a 3’–5’ direction and N7-guanine methyltransferase activity; Nsp 15, an EndoRNAse with Mn(2+)-dependent endoribonuclease function; Nsp16, a methyltransferase, modulating methylation of mRNA cap 2’-O-ribose to the 5’-cap structure of viral mRNAs [8]. The 3’-terminus of the viral genome includes four genes, encoding four structural proteins: the nucleocapsid- (N), the matrix- (M), the small envelope- (E) and the spike (S) glycoproteins and genes encoding accessory proteins. The N protein is enclosed in the core of the SARS-CoV-2 particle, interacts with the viral RNA and exerts a key role in the transcription of the SARS-CoV-2 genome and in nucleocapsid assembly to produce mature virions [9]. The S protein is composed of glycosylated proteins, forming spikes on the surface of viral particles and regulating the viral entry into the host’s cells [10]. The accessory genes are represented by the following: 3a, 3b, p6, 7a, 7b, 8b, 9b and 10 (Figure 1). These proteins inhibit type I interferon (IFN) synthesis and type I signaling pathways [11]. The structural and accessory proteins are generated from viral subgenomic RNAs (sgRNAs) [12]. The SARS-CoV-2 enters the human body via the airway tract, infecting both the epithelial cells of the trachea, bronchi, bronchioles and lungs, as well as resident, infiltrating and circulating cells of the IS [6]. This pathogen may directly invade the epithelial cells of the renal distal tubules, the cells of the mucosa of the intestine, the liver and the pancreas [13,14]. It may reach the neurons of the brain, the heart, the spleen, the lymph nodes and other lymphoid tissues [15]. In particular, the SARS-CoV-2 infects the host’s lymphocytes and macrophages, causing both their death through apoptosis and subsequent severe lymphopenia and, afterward, making them very efficacious carriers for its spreading [16,17]. Therefore, this virus reaches the mucosa-associated lymphoid tissue, bronchus-associated lymphoid tissue and nasopharynx-associated lymphoid tissue. The SARS-CoV-2 may induce a systemic involvement with different clinical courses. The genome of SARS-CoV-2 may be present in distinct sites of infected human hosts, including nasal cavities, pharynx, bronchoalveolar lavage, feces and blood [18]. The detection of live SARS-CoV-2 in blood samples positive by PCR tests and in feces suggests both a fecal route transmission and a host’s systemic infection [19,20]. Most of the patients may be asymptomatic or develop mild/moderate symptoms [21]. Only some subjects may undergo a severe disease requiring mechanical ventilation and admission to the intensive care units. In these cases, the onset of multiorgan dysfunction syndrome is associated with an elevated mortality [22]. The coordinated and tightly regulated activity of innate and adaptive arms of the IS is a crucial factor for the development of a protective response against viral pathogens invading the human body. Our knowledge concerning the dynamic changes of immune cells and of cytokines/chemokines patterns in patients with COVID-19 is still limited [23,24]. However, the available studies suggest that in the early phases of this disease, when the activation of the innate arm of the IS occurs, there is generally a suboptimal expression of type I and III antiviral IFNs and an increased release of other inflammatory cytokines, including mainly IL-6, MCP1, CXCL1, CXCL5 and CXCL10, also known as CXCL10/IP10 and TNF-α [25]. These mediators attract several immune cells, including polymorphonuclear leukocytes, monocytes, natural killer (NK) cells, dendritic cells (DCs), which also release further chemokines and generate the inflammatory process. DCs are antigen-presenting cells and represent a link between the innate and adaptive responses of the IS. These cells present SARS-CoV-2 antigens to specific T lymphocytes [26]. In particular, a recent study has shown that CD4^+^ and CD8^+^ T lymphocytes recognize multiple regions of the N protein in subjects convalescing from COVID-19. In addition, in individuals who were affected by SARS-CoV infection in 2003, a robust CD4 and CD8 T cell’s response against epitopes of the N protein of SARS-CoV was still detectable even 17 years later. These lymphocytes exhibited strong cross-reactivity toward the regions of the N protein of SARS-CoV-2 [27]. The involvement of lymphocytes marks the transition from the innate to the adaptive IR. This step is a key event in determining the course and the clinical outcomes of patients with COVID-19 [28]. In this phase, a favorable course of SARS-CoV-2-related infection depends on an efficacious activation of regulatory components of the IS, mainly mediated by specific CD4 and CD8^+^ T cells and by B cells. Under proper conditions, the development of plasma cells releasing specific and protective antibodies against the etiological agent of COVID-19 occurs [29]. An inappropriate and uncoordinated activity of the IS generates a robust inflammatory response, resulting in the cytokine release syndrome [30]. This clinical condition is characterized by severe clinical manifestations, including acute respiratory distress syndrome and disseminated intravascular coagulation [31]. In particular, the elderly patients show the most serious forms of SARS-CoV-2-related infections, where may be observed impairment in crucial activities of the IS, mainly in the T-cell-dependent responses [32,33]. Furthermore, elevated viral loads of this pathogen, assessed via the nasopharyngeal swab (NSWAB) samples taking advantage of the real-time RT-PCR, have been independently associated with higher MRs in comparison with individuals with lower SARS-CoV-2 levels [33,34]. An excessive proinflammatory Th1- and Th17-mediated response has been reported in the patients with SARS-CoV-2 infection, with increased concentrations of IFN-γ, TNF-α, IL-1β, IL-6, IL-15 and IL-17 [32]. In humans, T-helper 17 cells (Th17) are stimulated by IL-6 and IL-1β [35]. Studies ‘*in vivo*’ have suggested the potential proinflammatory role of some of them in the pathogenesis of SARS-CoV-2. Particular regions in nucleocapsid (N) and spike (S) proteins of this pathogen can directly bind several specific DNA sequences, detectable in the promoter region of a wide series of interleukins and cytokines, promoting the production and the release of these mediators [17,22,36,37]. SARS-CoV-2-induced disease with severe clinical courses and with a fatal outcome is characterized by a massive release of a wide spectrum of cytokines. This event is defined as ‘cytokine storm’ and leads to the life-threatening condition, known as ‘cytokine release syndrome’ [38]. The cells (macrophages, monocytes, lymphocytes and DCs), the mediators, costimulatory-, inhibitory-molecules and the mechanisms involved in this complex process have been widely described in recent studies [17,22,39].

### HIV structure & pathogenesis

**Figure 2.**
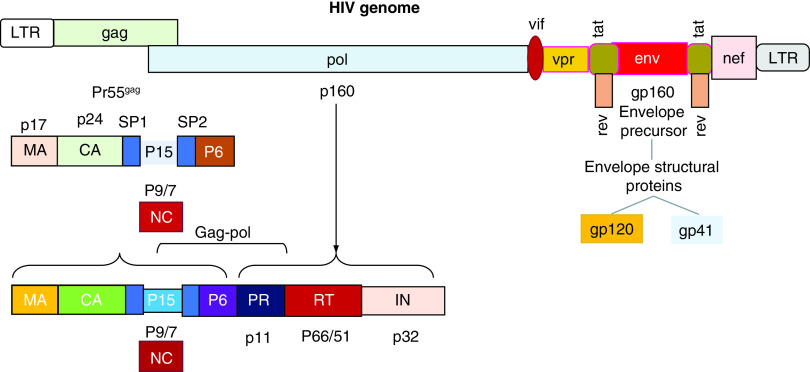
HIV consists of two copies of positive-sense ssRNA, housed in a conical capsid. HIV genome is composed by at least nine genes, defined *gag, pol, env, tat, rev, nef, vif, vpr, vpu*, encoding 19 proteins and two LTR regions at their 5′- and 3′-ends. Gag, pol and env codify structural proteins, which are incorporated into the new virions. Env encodes a precursor glycoprotein (gp160), which is cleaved into two parts, defined gp120 and gp41, by a cellular protease. Three molecules of gp120 and three of gp41 form a structure anchoring to the viral envelope. This apparatus allows gp120 to interact with specific receptors on target cells, such as CD4. The *Gag* (group-specific antigen) gene encodes the information for the synthesis of a 55-kDa polyprotein. This molecule is cut into several proteins after viral budding, including MA, CA, NC, spacer p1, spacer p2 and p6 domain. The *pol* gene encodes the enzymatic PR, RT and IN. *Tat, rev, nef, vif, vpr* and *vpu* represent regulatory genes and express proteins, modulating and regulating viral infectivity and replication and affecting the HIV ability to induce disease in the infected host (see text). CA: Capsid; IN: Integrase; LTR: Long terminal repeat; MA: Matrix; NC: Nucleocapsid; PR: Proteins protease; RT: Reverse transcriptase.

HIV is a spherical-shaped nonsegmented virus with a diameter of about 120 nm. It consists of two copies of positive-sense ssRNA, housed in a conical capsid. The viral genome sequence is approximately 9200–9600 bases in length [40]. According to the phylogenetic analysis, HIV consists of two major types, HIV type 1 (HIV-1) and HIV type 2 (HIV-2). HIV-1 is divided into four different groups: M (major), O (outlier), N (non-M, non-O) and P (pending) [41]. The viruses belonging to Group M represent the cause of the AIDS pandemic worldwide and have been subdivided into nine subtypes, designated by the letters A–D, F–H, J and K [42]. The conical capsid structure is surrounded by a matrix and by a more external element, represented by a viral envelope (VE). Both elements together contribute to the maintenance of the virion integrity. The VE is composed of a lipid bilayer, derived from the plasmatic membrane of infected cells when the virions bud from them [43]. HIV genome includes at least nine genes, known as *gag, pol, env, tat, rev, nef, vif, vpr, vpu* and codifying 19 proteins and two long terminal repeat regions (LTRs) at its 5’- and 3’-ends. *Gag, pol* and* env* encode structural proteins, which are incorporated into the new virions [44]. *Env* codifies a glycoprotein (gp)160, which is cleaved into two parts, defined gp120 and gp41, by a cellular protease. Three molecules of gp120 and three of gp41 form a cap and a stem respectively, generating a structure anchoring to VE. This apparatus allows gp120 to interact with specific receptors on the target cells, such as CD4 [45]. Changes in gp120 and gp41 structural conformation occur with the reorganization of their spatial disposition and the exposition of coreceptor-binding domains. The physical interaction between HIV and specific cell receptors induces the fusion of the VE with the plasmatic membrane of target cells. The content of HIV virions enters the host’s cells, initiating a productive infectious cycle. The *Gag* (Group-specific antigen) gene encodes the information for the synthesis of a 55-kDa polyprotein in the cytoplasm of infected cells. This molecule is cut into several proteins after viral budding: Matrix (MA), Capsid (CA), Nucleocapsid (NC), spacer p1, spacer p2 and p6 domain. About 1500 copies of the HIV protein CA monomers form the structure of the viral conical capsid, whereas the matrix surrounding it consists of MA elements. A ribonucleoprotein containing NC complexed to genomic RNA in association with the replicative enzymes forms the inner component of each virion. The *pol* gene encodes the enzymatic proteins protease (PR), reverse transcriptase (RT) and integrase (IN). HIV complete virions, causing a productive infection in the target cells, include these three enzymes. Viral RNA is converted into DNA by RT and inserted into the DNA of the host’s cell nucleus, where it may either remain in a latent status or undergo the process of transcription into messenger RNA and generate the viral polyprotein. Some complex events characterize HIV replication. In particular, the mature PR cut this polyprotein into the prior indicated proteins. The PR precursor generates its functional form from the polyprotein using a mechanism defined autoprocessing. This event consists of a double cleavage involving both its N- and C-terminus [46–48]. *Tat, rev, nef, vif, vpr, vpu* represent regulatory genes and express proteins, modulating and regulating viral infectivity and replication, and allow HIV to induce disease in the infected host [49]. In particular, *Tat* is a multifunctional protein, expressed into two forms (p16 and p14) and synthesized during the early phases of the viral cycle. *Tat* influences chromatin remodeling, phosphorylation of RNAp II, modulating the transcription of the full-length viral mRNAs in transactivation of viral genes and in interaction with specific sites of HIV-1 mRNAs. *Tat* proteins considerably increase the efficiency of HIV replication [50]. In the cells, the *rev* protein (p19) is involved in a crucial process of HIV replication, as it regulates the export of unspliced RNA messengers from the nucleus to the cytoplasm in the infected cells. The lack of *Rev* protein is associated with the retention in the cell nucleus of RNA messengers codifying structural viral genes (*Gag* and *Env*). The *nef* negative regulatory factor physically binds to a wide series of the components belonging to the host’s molecular machinery in the cytoplasm. Nef controls protein-trafficking paths and promotes the release of virions and increases their infectious ability. The *vif* (virion infectivity factor) (p23) is a protein blocking a cellular cytosine deaminase, known as APOBEC3G. This enzyme represents a constitutive cell defense, converting cytidine into uridine and causing alterations in the viral genome during reverse transcription. Viral particles in cells expressing APOBEC3G will consequently not be able to replicate. The *vif* protein interacts with APOBEC3G and forms a complex, binding ubiquitin, thereby causing the proteolytic degradation of this enzyme. It is also a system to neutralize one of the organism’s innate defenses against viruses. The *vpr* protein or viral protein r (p14) modulates the entry of the HIV-1 preintegration complex into the nucleus, regulates its replication in nondividing cells including macrophages and causes the cell cycle arrest in the G2 phase and in the apoptosis in proliferating cells. The *vpu* protein or viral protein u (p16) promotes the degradation of CD4 receptors in the ER and increases the release of HIV virions [49]. The organization of the HIV genome is shown in Figure 2.

HIV infects cells expressing the CD4 receptor and the chemokine receptors CCR5 and CXCR4, including CD4^+^ T cells; Monocytes and macrophages (CD68^+^), detectable in lymph nodes, spleen, liver, brain, lung, bone marrow; DCs in lymphoid germinal components and in lymphoepithelial structures, detectable in the vagina, tonsils and rectum. HIV may infect cells in a wide spectrum of human tissues through independent CD4 receptors, including kidney tubules, astrocytes, myocardiocytes, enterocytes and endothelial cells [51,52]. Nowadays, it is possible to identify HIV-infected patients during the first weeks, knowing that the acute phase of this disease elapses about 3–6 months. During this period, after its entry in the host’s IS HIV is activated, with the increase of a wide spectrum of plasma cytokines/chemokines [53]. Stacey *et al.* have studied the dynamic pattern of these mediators in samples obtained from patients with acute HIV-, HBV- and HCV infections during the earliest phases of exponential viremia increase. An elevation in the levels of these mediators has resulted in all the groups, but with distinct hierarchy and kinetics, depending on the different viruses. In particular, the viral expansion in subjects with acute HIV-1 infection has been characterized by dynamic changes in plasma levels of several cytokines/chemokines, such as the fast and the transient increase in IL-15 and IFN-α, the long-lasting elevations in IP-10, in TNF-α and in MCP-1, as well as the slow rise of IL-6, IL-8, IL-18, IFN-γ and IL-10. The rapid activation of the cytokine pathway, observed in this pathological condition, originates the process known as a cytokine storm. However, the magnitude of this strong systemic cytokine release is unable to control HIV infection and to prevent its persistence [54]. Similar results have been obtained in a research led by Muema *et al.*, who have investigated the kinetics of plasma cytokines, immune cell dynamics and viral replicative ability in a cohort of patients with hyperacute HIV infection before the viral expansion has reached its peak. This study has confirmed the increase of plasma IP-10, MIG, IFN-γ, IFN-α, MCP-1, IL-12, soluble IL-2 receptor, IL-1RA, IL-8, CXCL13 and soluble CD14 in untreated individuals. In these subjects, acute HIV infection is associated with the development of cytokine storm, with lymphocytes-, eosinophils- and basophils depletion, with a rise in the monocytes of peripheral blood [55].

### HCV structure & pathogenesis

**Figure 3.**
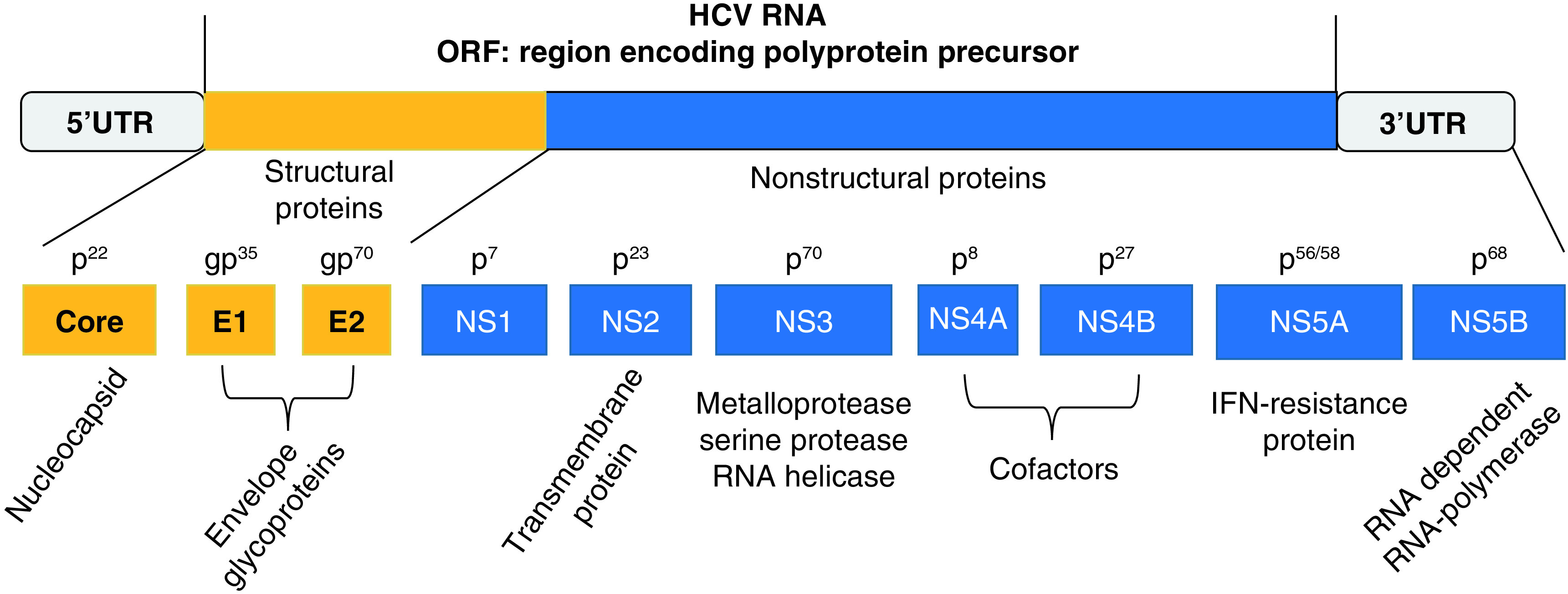
HCV consists of a ssRNA genome, approximately 9.6 kb in length. The viral genome includes a single ORF codifyng a polyprotein precursor of about 3010 aminoacids in length and 5′- and 3′-UTRs at both ends. Polyprotein cleavage generates mature structural- (core, E1 and E2) and nonstructural (NS1 or p7, NS2, NS3, NS4A, NS4B, NS5A, NS5B) proteins. Core **(C)** protein is involved in the development of viral nucleocapsid structure and by the envelope glycoproteins; E1 and E2 are detectable on cell membrane; NS1or p7 is a short membrane protein; NS2 is a transmembrane protein; NS3 protein has serine protease- and NTPase/helicase activity; NS4A protein has a double function: cofactor for NS3 protein, its binding to NS3 increase its activity with a more efficient process of cleavage; modulation of NS5A phosphorylation; NS5A with unknown function; NS5B is the RdRp. ORF: Open-reading frame; RdRp: RNA-dependent RNA polymerase; UTR: Untranslated region.

HCV is a small circular enveloped positive-sense, single-stranded nonsegmented RNA genome virus, approximately 9600 bases in length [56]. Viral particles are about 50–70 nm in diameter [57]. HCV belongs to the genus of *Hepacivirus* and to the family of Flaviviridae [58]. Its genome includes a single ORF codifying a polyprotein of about 3010 aminoacids in length and 5′- and 3′-untranslated regions (UTRs) at both ends (Figure 3). These UTRs are highly conserved key RNA elements for a proper viral genome replication and protein translation. A 340-nucleotide long sequence at 5′-nontranslated region (NTR) functions as an internal ribosome entry site. When this zone in the HCV genome binds the 40 S ribosome subunit, the process of the polyprotein synthesis in the host’s cells starts [59]. Cell- and virus-associated proteases cleave HCV polyprotein at the ER membrane, generating ten mature structural and nonstructural proteins. The former group of proteins is represented by the core (C), involved in the development of viral nucleocapsid structure and by the envelope glycoproteins E1 and E2. These proteins are detectable on the surface of virions and are essential in mediating the HCV entry into the host’s susceptible cells via the binding to specific cell membrane receptors. The latter group includes: a short membrane protein, the peptide p7 or NS1; the NS2 protein, a transmembrane protein; the NS3 protein, with serine protease activity at its N-terminal and an NTPase/helicase activity at its C-terminal; NS4A protein, a cofactor for NS3 protein, binding to NS3 increases its activity and promotes a more efficient process of cleavage; NS4A also modulates the phosphorylation of NS5A; NS5A is a polyphosphorylated protein with unknown function; NS5B is the RdRp [59]. NS3 serine-like protease and the RdRp are believed to be components of the HCV replication complex and are required for efficient viral maturation and replication. In particular, HCV core proteins interact with viral RNA and generate nucleocapsid particles on the membrane of the ER in the host’s cells. Then, these particles associate with VE proteins, embedded in the ER membrane, generating complete enveloped virions by budding into the ER lumen. RdRp lacks of proofreading activity and so the HCV genome is characterized by remarkable sequence variation. To date, seven HCV genotypes have been described with a wide number of subtypes [60]. HCV generates within the infected hosts a pool of genetically different variants, although closely related among them. They are known as ‘quasispecies’. HCV virions are detectable in different forms in the serum of human carriers. In particular, viral particles may either bind to low-density and very low-density lipoproteins, or to immunoglobulins [61]. HCV may circulate in the blood in the form of free virions [62] and infect not only liver cells but also peripheral blood mononuclear cells (PBMNC), including T-, B-monocyte and macrophage populations [63] and even cells of extrahepatic tissues, such as heart, kidney, pancreas, intestine, adrenal, lymph node and gallbladder. HCV antigens, HCV-RNA and its intermediate forms have been detected in the cells of all these organs, suggesting that these sites can act as a reservoir for this pathogen and may host its replication [64]. A large series of studies concerning HCV acute infection in humans and chimpanzees have shown that this condition may be followed by a double outcome: either the spontaneous clearance of this pathogen or its persistence in the host. The former event is characterized by the generation of a pool of HCV-specific CD4 and CD8 T cells [65]. These T cells release a wide spectrum of cytokines and exert strong and sustained effect on polyfunctional activities against multiple epitopes within the different proteins of this pathogen both in intrahepatic and peripheral sites [66]. This type of IR leads to the control of HCV and to the development of specific memory T cells, which express the IL-7 receptor CD127 on the plasmatic membrane [67]. If the IS fails in controlling HCV infection, specific CD8 T cells undergo exhaustion and increase the expression of some specific markers, including PD1, Tim-3, CTLA4, 2B4, CD160, KLRG1, TIGIT and CD39 [68]. Moreover, the production of early neutralizing antibodies can entail a favorable impact on the outcome of the acute infection [69]. According to the results of Stacey *et al.*’s study, a rapid increase in plasma concentration of proinflammatory cytokines and chemokines, including TNF-α, IFN-γ, IL-6, IL-10, IL-18, IP-10, MIP-1 β and CCL-5, was observed in patients with acute HCV infection during the earliest phases of the disease. However, in this group the production and the release of the above-mentioned mediators have been delayed and of lower magnitude in comparison with the ones observed in individuals with HIV-related acute infection. Other substantial differences have been observed in the hierarchy of cytokines/chemokines elevation, detectable in acutely HIV- and HCV-infected patients [54,70]. According to Stacey *et al.*’s conclusions, two important points have to be underlined: the activation of the cytokine cascade in subjects with acute HCV infection does not reach such a magnitude as to originate the process known as a cytokine storm described in patients with HIV infection; even if some of these cytokines/chemokines may play a role in the control of HCV replication, an excessive and uncoordinated release of these mediators is not protective and curative for the infected host. This event is rather associated with both the well-known acute and the long-lasting immune-pathological consequences deriving from this infection.

### IVs structure & pathogenesis

**Figure 4.**
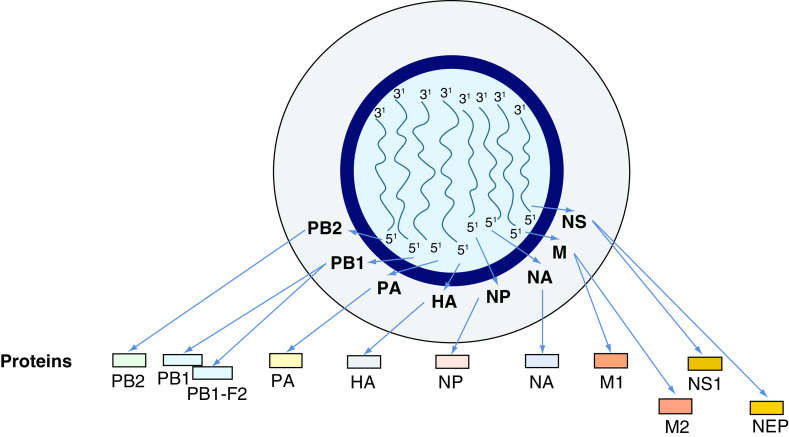
Representation of influenza virus genome. Humans may be infected by influenza viruses type A, B & C. They differ in their organization, but only the A and B ones cause important clinical manifestations in mankind. Type A is considered as a paradigm. Influenza virus genome is about 13.5 kb in length, consists of a ssRNA with negative polarity (-) ssRNA and includes eight ssRNA segments. Each of these elements is separately enclosed in vRNPs. The 5′- and 3′-termini of viral RNA are associated with RdRp with multiple copies of NP. The influenza virus genome is composed of 11 proteins (HA, NA, NP, M1, M2, NS1, NS2 or NEP, PA, PB1, PB1-F2, PB2). The RNA polymerase of the influenza virus consists of three subunits, such as PB1, PB2 and PA, codified by three genes in influenza A and B viruses. A matrix consisting of M1 protein includes the virion core and it is surrounded by an external lipid envelope, where three integral glycoproteins, such as M2 (an ion channel), HA and NA are embedded. HA and NA are used to classify the different serotypes of influenza A viruses. Additional influenza virus proteins include the accessory protein PB1–F2, displaying proapoptotic activity, NS1 protein with anti-IFN-β function and promoting of viral replication, NEP (or NS2) nuclear export of viral ribonucleoproteins. HA: Hemagglutinin; NA: Neuraminidase; NP: Nucleoprotein; PA: Polymerase acidic; PB1: Polymerase basic 1; RdRp: RNA-dependent RNA polymerase; vRNP: Viral ribonucleoprotein complex.

IVs type A, B and C belong to the family of Orthomyxoviridae and possess the capacity to infect mankind, but only the A and B ones, provoking important clinical manifestations in humans [71]. All these types of IVs consist of a ssRNA with negative polarity ([-] ssRNA), but they differ in their organization [71]. By electron microscopy, IVs A and B appear as spherical or filamentous forms, about 100 nm in diameter and 300 nm in length, respectively. The genome of both viruses is about 13,500 bases in length with eight ssRNA segments. Each of these elements is separately enclosed in viral ribonucleoprotein complexes. Within these structures, the 5′- and 3′-ends of viral RNA are associated with RdRp with manifold copies of nucleoprotein (NP). The viral ribonucleoprotein complexes regulate the viral life cycle, modulating transcription and replication of this pathogen in infected cells [72]. The IVs genome encodes 11 proteins (HA, NA, NP, M1, M2, NS1, NS2 or NEP, PA, PB1, PB1–F2, PB2; Figure 4) [73]. The RNAp of the IVs is composed of three subunits: polymerase basic 1 (PB1), PB2 and polymerase acidic (PA) in IVs A and B. A matrix consisting of M1 protein incorporates the virion core and it is surrounded by an external lipid envelope, where three integral glycoproteins, such as M2 (an ion channel), hemagglutinin (HA) and neuraminidase (NA), are embedded [74]. The several subtypes are indicated according to an H number (for the type of HA) and an N number (for the type of NA). To date, 18 different H antigens (from H1 to H18) and 11 distinct N antigens (from N1 to N11) have been identified [71]. HA is a lectin mediating the entry of the viral genome into target cells, while NA is an enzyme involved in the release of the virions from infected cells [75]. These two glycoproteins are both a target for antiviral drugs and for specific antibodies [76]. Therefore, they are used to classify the different serotypes of IVs A (16 HA and nine NA) [71,77]. The isolated influenza strains are identified through a standard nomenclature indicating the type of virus, the geographical location where it was first isolated, the sequential number of isolation, the year of isolation and the HA and NA subtypes [71]. Additional IV accessory proteins include: PB1–F2, with proapoptotic activity, NS1 with anti-IFN-β and with proreplicative functions as well as NEP (or NS2), promoting nuclear export of viral ribonucleoproteins. IVs are mainly transmitted via the air and spread very easily through the droplets of saliva that the patient produces by coughing, sneezing or simply talking, especially in crowded and closed environments [78]. New IVs constantly emerge by mutations or reassortment in their genome, leading to the events, respectively, known as ‘antigenic drift,’ with the generation of ‘strain variants’ and as ‘antigenic shift’ when the virus acquires completely new antigens by recombination between avian- and human-viral forms. One of these variants may, eventually, achieve a greater virulence becoming dominant and rapidly spreading throughout the population and often causing a pandemic [79,80]. According to an alternative model, periodic pandemics are produced by the interaction between a fixed set of viral strains and a human population with a constantly evolving IR against these pathogens [72,80–82]. High levels of several proinflammatory mediators including IFNs, TNF-α and IL-1β, IL-6, IL-8, IL-10, MCP-1, IP-10 and monokines induced by IFN-γ (MIG) have been observed in the patients admitted to hospitals with severe forms of IVs infection (2009 H1N1 and H5N1) and have been hypothesized to be present in subjects who suffered from H1N1-related disease in 1918 [83,84]. The cytokine storm has been described also in patients infected by these pathogens [85,86]. Elevated plasma amounts of these interleukins/cytokines are thought to be strongly associated with the severity of the disease, exerting a direct role in morbidity and mortality [87,88].

## Considerations on the onset & spreading of pandemics caused by respiratory viruses according to a historical perspective

Since the 19th century, several viral pandemics, almost caused by IVs, spread across the world, generally during the autumn and the winter months [89–91]. In this seasonal period, cold, decreased hours of exposure to sunshine, elevated levels of humidity and crowding of individuals in closed spaces are optimal conditions to promote the spread of epidemics associated with respiratory pathogens. The major viral pandemics generally start in Eastern or Southern Hemisphere nations and then tend to disseminate to Europe and to America [92]. During the course of these infectious events, the number of affected individuals and of deaths is characterized by multiple well-separated temporal peaks with a time-scale of months [93]. Each of these epidemics showed its own features, including the type of viruses involved, transmissibility, geographical area of onset, clinical impact in distinct regions of the globe and the death rate in the general population or in the different age-classes. Overall, these infectious events occurred with different waves over a duration of 2–3 years. The main characteristics of each epidemic are shown in Table 1 [94–106]. To date, the mechanisms causing single or multiple waves during pandemics are not definitively clarified. Some researchers have proposed mathematical models to quantitatively reproduce the pattern of the waves and to recreate the course of the outbreaks during the pandemics [107]. One study has examined the conditions contributing to generate two-waves-shaped curves via the design and the analysis of five possible mathematical models. This strategy simulates the development and the course of acute IDs as observed in the actual clinical practice [107]. The ‘Russian pandemic influenza’ began in October 1889, reaching Europe and North America in 1890 [104], with distinct recurrences in March–June 1891, November 1891–June 1892, winter 1893–1894. The second wave occurred during the spring of 1891 and the third one in the winter of 1892 resulted more lethal than the first wave, with about 1 million of deaths. Most of them were caused by respiratory forms, mainly in the middle-age patients [92,100]. Three different viral strains have been proposed to be as possible agents responsible for this infection, including IVA subtypes H2N2 or H3N8 or the human coronavirus OC43 [104,106]. Also during the Spanish pandemic four waves occurred [98,103]. The first one in March 1918, the second in the half of August 1918, while World War I was ongoing, the third and the fourth ones in the period January–June 1919 and January–April 1920, respectively [108]. Even during this infectious outbreak, the second and third waves resulted more lethal than the first one [109]. Although the number of deaths registered during this pandemic remains uncertain, it has been calculated that 500 million–1 billion people became infected and about 17–50 million individuals died [89]. This pandemic was caused by the IVA, subtype H1N1. Mortality rates (MRs) were elevated in individuals younger than 5 years old, as well as in subjects 20–40 years old and 65 years and older. The high mortality observed in the healthy population, including people in the 20–40-year old class, represented a unique feature of this pandemic [103,110]. The Asian influenza pandemic onset occurred early 1956 or 1957, probably in Guizhou, where the first cases were observed [105]. Then, it spread to Yunnan province and to Hong Kong on April 1957, reaching Singapore, Taiwan, Japan and India in May. In June 1957, the epidemic involved the UK and the USA. In the USA, the first phase of the outbreak mainly affected children returned to school after the summer break in 1957 [102]. This circumstance probably provided the opportunity for the pandemic to spread in this group of patients with the development of high morbidity and MRs. In the following period (January–March 1958), the MRs of children decreased, whereas they remained elevated in the age groups of 45–74 years old or of over 75 years old [111]. During the second wave, about 500 million individuals were infected worldwide, with more than 1 million deaths. The IVs A subtype H2N2, generated by the reassortment of avian and human influenza viruses, caused the Asian influenza pandemic. After the first two waves, two major recurrences of the epidemic occurred in January–March 1960 and in January–March 1963. These outbreaks were caused by H2N2 viral subtypes, progressively acquiring minor genetic modifications (an event known as antigenic drift). Then, after a decade, a new IVA subtype emerged (H3N2 strain) and was associated with the development of the Hong Kong pandemic in 1968 (an event known as antigenic shift) [96,112]. The genome of this pathogen included two genes, originating from an avian influenza virus and from six genes from the IVA (H2N2), which spread across the world during the 1957 infectious event [113]. The 1968 pandemic was characterized by two waves, the first one in 1968/1969 and the second one in 1969/1970. The last outbreak manifested with a drift in the NA antigen [114]. Although it has been hypothesized that the onset of the 1968 pandemic had occurred in Mainland China, the first case was observed on 13 July 1968 in Hong Kong. At the end of the month, the virus reached Vietnam and Singapore and in September the Philippines, the northern Australia and Europe were involved. In December 1968, the pandemic spread in the USA and in 1969 in Japan, Africa and South America. A peak in the number of deaths was reached in December 1968 and in January 1969 across the globe [94,97]. Most of the deaths associated with this infectious event (about 70%) were observed in 1968/1969 in North America (the USA and Canada). In Europe, Japan and Australia the majority of the subjects (70%) died during the second wave of the epidemic in 1969–1970. The causes of this difference in MRs detected in North America and in Europe, Japan and Australia are not well understood. However, a phylogenetic analysis of the virus found a drift in the NA antigen, but not the HA antigen [114]. Lindstrom *et al.* have shown that two distinct NA lineages were identified during the 1968/1969 and the 1969/1970 pandemic, and suggested that this genetic diversity could have been caused by several reassortments occurring in A/H2N2 viruses. This evidence might contribute to explain the differences in disease severity and in clinical outcomes observed in distinct geographical areas in the two considered periods (1968/1969 and 1969/1970) [115]. It has been calculated that at least 500 million individuals were infected worldwide and more than 1 million deaths occurred [116]. The 2009 Swine IV is an H1N1 strain, defined as H1N1/09. It originated from the recombination of genomic material from bird, swine and human flu viruses with a Eurasian pig flu virus. The epidemic developed between January 2009 and August 2010, starting from Mexico, then it spread worldwide [101]. This pandemic was characterized by at least two waves, although some researchers have reported the third one. The first infectious event occurred in April–July 2009 and, according to a research carried out in the UK, it quickly subsided at the onset of the summer closing period for schools. The second wave appeared in August 2009 when the students resumed school lessons and lasted until March 2010 [95]. A third outbreak occurred between December 2010 and February 2011, and was described in several countries, including Denmark and the UK, where this infectious event was associated with an atypically cold period with dry weather [95,117]. Both the second and the third waves were characterized by a more severe clinical course in the affected patients in comparison with the first one [118]. However, to date, it has not been demonstrated that the potential increased virulence of this virus, as observed in 2010 and in 2011, is correlated with the emergence of viral clusters, which have undergone the event known as antigenic drift in viral 2009 progenitor or with the development of further mutations in its genome [99]. Overall, near 80% of deaths associated with the Swine influenza virus were observed in people younger than 65 years of age. According to CDC estimates, about 151,700–575,400 people across the globe died from the H1N1/09-related infection during the first year of the pandemic [119,120]. Overall, the available historical descriptions, as well as the results of the more recent studies focusing on the previous viral epidemics (Russian-, Spanish-, Asian-, Hong-Kong-, Swine Flu epidemics), teach to us that each of these infectious events consists of peculiar signs in comparison with typical seasonal flu outbreaks as it is generally associated with at least two or three waves, with a more elevated patients’ morbidity and mortality during the second and third waves in comparison with the first one, as well as with possible recurrences connected to the event known as antigenic drift [96,101,104]. Therefore, these observations must be taken into strict consideration by the governments and the health services of each nation across the world and should suggest planning preventive programs with the purpose to hamper the deleterious effects of the current and of possible future SARS-CoV-2-related waves. The uncertainty of controlling the development and the spread of a new ID leads us to evaluate the possible effectiveness of health measures already applied in the past during previous pandemics with beneficial results. Although influenza viruses-related pandemics are characterized by peculiar signs in comparison with typical seasonal flu outbreaks, such as numbers of waves, generally more than two/three, morbidity and MRs, some important lessons and common elements can be drawn by analyzing the onset and the development of the different pandemics. In particular, some papers have performed historical investigations concerning the public health measures adopted during the past pandemics, with the purpose to obtain useful informations for the management of the current outbreak. Some researchers have also compared the healthcare strategies implemented during the 1918 influenza pandemic, which has been considered as a paradigm, and the ones carried out up to now during the spread of SARS-CoV-2 infection in different countries worldwide [121]. To date, a lot of general prophylactic strategies, proven to be effective in the course of Spanish flu and in other epidemics, have been reintroduced in order to counteract COVID-19 diffusion. These healthcare interventions include actions for increasing the health education of people to improve the knowledge about the characteristics of this virus and the mechanisms regulating its infectivity and its capability to spread into the population across the globe. The isolation of infected patients and social distancing among individuals, the improvement of hygiene habits, with the use of facial masks and with the promotion of hand disinfection by means of hydroalcoholic preparations and the strict surveillance of subjects who have come into contact with patients suffering from COVID-19, have been widely suggested and performed during this pandemic. The quick implementation of some general prophylactic measures has contributed to decrease the number of infections and deaths both during the period of Spanish flu and today at the time of SARS-CoV-2 outbreak, but some problems still remain, although at least a century has elapsed after the influenza virus pandemic. Some researchers have reported some degree of unpreparedness in the management of COVID-19, not only in general population but also in central and peripheral authorities. Several differences in the adoption of preventive strategies against SARS-CoV-2 have emerged worldwide among Scientific Committees, Chiefs of Government and Minister of health, due the high risk of the onset of socioeconomic crisis caused by the prolonged measures of lockdown and by the limitation of individual freedom [121,122]. Moreover, the current pandemic has proposed again a problem already observed in 1918. Several crucial disparities still remain among high-income and low-income countries, as well as in the developed nations among racial groups or among rich or poor individuals. These ineuquities have been evidenced in the access to healthcare services, in the therapy and in the availability of preventive strategies, such as vaccination against this pathogen [121,123].

**Table 1.** Main characteristics of Russian-, Spanish-, Asian-, Hong-Kong-, Swine influenza pandemics, including the virus strain involved, the geographical area of onset, the clinical impact in distinct regions of the globe and the death rate in the general population or in the different age classes.

Name of pandemic	Virus strains	Date of onset/duration	Waves/period of onset (n)	Suspected origin of epidemic	Estimated world population	Estimated infected population	Deaths in the world (n)	Case fatality rates	Ref.
‘Russian’ pandemic influenza	Likely H3N8 or H2N2 Or Corona-virus OC43	1889 1889–1894	4 First: October 1889–December 1890; Second: March–June 1891; Third: November 1891–June 1892; Fourth: winter 1893–1894	Russia	1.5 billion	300–900 million	1 million	0.1–0.3%	[100,104,106]
‘Spanish’ pandemic influenza	H1N1	1918 1918–1920	4 First: March 1918 Second: second half of August 1918 Third: January–June 1919 Fourth: January–April 1920	China	1.8 billion	500 million–1 billion	17–50 million	2–3% Until 10%	[96,98,103]
Asian influenza	H2N2	1957 1957–1958	2 First: October–December 1957 Second: January–March 1958 Recurrences of H2N2 virus associated pandemic in: January–March 1960 January–March 1963	China	3 billion	About 500 million	1.1 million	0.2–0.3%	[95,102,105]
Hong Kong influenza	H3N2	1968 1968–1970	2 waves First: July 1968–April 1969 Second: November 1969–March 1970	Hong Kong	3.5 billion	About 500 million	1–4 million	0.1–0.3%	[94,97]
Swine influenza	H1N1/09	2009 2009–2010	2/3 waves First: April–July 2009 Second: August 2009–March 2010. Third: November/December 2010–February 2011	Mexico	6.8 billion	About 700 million–1.4 billion	151,000–575,000	0.01%	[99,101]

## Considerations on the possible establishment of a persistent SARS-CoV-2 reservoir worldwide & on the potential risk of developing malignant & nonmalignant conditions

To date, it is thought that at least 178,000,000 people in the world have come into contact with the virus with about 3.8 million of deaths (WHO, Coronavirus Disease COVID-19 Dashboard, accessed on 19/06/2021), but this number could be underestimated. Based on the observation that the capacity to record the number of deaths, associated with SARS-CoV-2 infection, widely and remarkably differs both across and within the regions worldwide and on the assumption that it may be many times higher than one officially reported, the Institute for Health Metrics and Evaluation has recently provided an estimation of total mortality due to COVID-19. This discrepancy may depend on some reasons. In particular, the counting of the demises may have been or may be underestimated both in developed and in low-income nations, due to the difficulties in identifying all the deaths associated with SARS-CoV-2 infection. Therefore, an undefined proportion of these fatal events may remain unrecordered. Plus, since the beginning of 2020, a significant difference, generally known as ‘excess mortality’, has been reported and is underlined in yellow in several countries worldwide between the observed death rates and the expected ones. The researchers of the Institute for Health Metrics and Evaluation have developed accurate models for the statistical analysis of the above reported data and variables and have predicted the ratio of total COVID-19 mortality to reported COVID-19 mortality for all the regions worldwide in the period ranging from March 2020 to 13 May 2021. According to the figures achieved and despite a high level of heterogeneity concerning the data obtained across the different countries considered, it has been estimated that about 7.1 million individuals have died worldwide owing to SARS-CoV-2 infection since the beginning of the pandemic. On the other hand, the officially reported number of deaths worldwide in the same period is equal to about half of this estimation, with nearly 3.4 million demises [2]. Understanding when a patient with a proven positivity to specific tests for detecting the presence of a virus has cleared the pathogen represents a crucial element for the control of IDs. According to the initial WHO’s recommendations, subjects with a previous diagnosis of SARS-CoV-2-related infection are confirmed to have cleared the virus and may be discharged from isolation when they are clinically recovered and have two negative RT-PCR results on sequential samples taken at least 24 h apart [124]. RT-PCR test is currently considered the gold standard for the diagnosis of SARS-CoV-2 infection. An update of the criteria for the conclusion of the isolation as part of the clinical care in patients with SARS-CoV-2-related infection has been proposed on 27 May 2020. The application of these recommendations concerns all the patients suffering from this disease irrespective of the affected areas worldwide or the disease severity [124]. Criteria for the discharge of the subjects from isolation without requiring retesting are: in symptomatic patients, 10 days following the development of symptoms plus at least three additional days without symptoms (in absence of fever and without respiratory symptoms); in asymptomatic patients, 10 days following positive tests for SARS-CoV-2 [124]. Although on the basis of these recommendations, subjects with a previous contact with this pathogen are considered to have cleared SARS-CoV-2, there is still the possibility that this pathogen may constitute a persistent reservoir at least in a part of the very large number of people who had a contact with this pathogen worldwide. This potential biological behavior for SARS-CoV-2 is not surprising, as it has been observed for other viruses-related diseases, not only in subjects receiving chemotherapy or immunosuppressive therapy but also in apparently immune competent individuals, although at lower rates throughout the patient’s lifetime. It has been shown that both RNA and DNA viruses have elaborated mechanisms to establish latent or occult infections in human hosts. These pathogens are represented by HIV, HCV, respiratory syncytial virus (RSV), Measle virus (MV), West Nile virus (WNV), Zika virus (ZV), Entero virus (ENV) and even Ebola virus (EV) [125]. In addition, this condition of persistence is associated with a risk of viral reactivation. RNA viruses, such as HIV and HCV, exhibit this biological behavior [126–128]. Even if the contribution of RSV, MV, WNV, ZV, ENV and EV to the long-term human pathology has not been defined yet, some patients who were infected by these pathogens experienced complications or diseases lasting for weeks or months after the acute phase of infection and the genome of these pathogens was detectable for the same period of time [125]. It has been observed how DNA viruses, such as herpesviruses, adenoviruses, polyomaviruses and HBV can establish lifelong persistence infections. Among these pathogens, HBV and Herpesviruses are able to induce the development of severe sequelae in a significant percentage of infected individuals, and herpesvirus family present this biological behavior [129–131]. It is now well known that HCV-RNA and HBV-DNA can be present and detectable in the liver and/or cells of different tissues, such as immune, pancreatic and renal cells, even in subjects without a well-recognized and well-recorded contact with these pathogens or in individuals who have reached the apparent eradication of these viruses after their spontaneous or therapy-related clearance. This condition is known as occult infection and it has been described for HCV, as well as for HBV [130–132]. HIV also displays strategies to induce its persistence in human hosts, such as promoting a latent stable reservoir in cells expressing the CD4 receptor, such as resting memory CD4(+) T cells, monocytes and macrophages (CD68^+^), as well as dendritic and also in cardiac muscle cell, endothelial cell, kidney tubules cell, astrocytes and enterocytes [51]. This condition contributes to facilitate its persistence in infected subjects and to promote its transmission to other individuals [127–133]. Several sites of latency have been also described for the members of the herpesvirus family. EBV infects human lymphocytes and oropharyngeal epithelial cells, whereas herpesvirus-8 colonizes monocytes as well as endothelial- and B-cells. Primary varicella-zoster virus establishes a lifelong latent infection in ganglionic neurons. Its reactivation in infected individuals induces a disease known as Herpes Zoster, associated with neurological complications [134–136]. According to these observations, it is possible that even SARS-CoV-2 may become persistent in infected cells. This aspect will be of great relevance in clinical practice and will have to be investigated in the future in order to clarify the possible carcinogenic properties of this virus and its potential impact on human nonmalignant as well as malignant pathologies. This assessment will be important also in patients who have been reported to have cleared SARS-CoV-2 and to have obtained a complete recovery. HCV, HIV and HBV are oncogenic pathogens and they have been associated with the development of noncancerous diseases, other than with several types of cancers. A broad spectrum of long-term HCV-related hepatic and extrahepatic manifestations, both malignant and nonmalignant ones, has been described in the literature. Several organs are involved by this pathogen [137,138]. HCV persistent infection is characterized not only by mild to severe forms of liver damage but also by a wide spectrum of extrahepatic manifestations, ranging from conditions with subclinical expression to the ones with very serious clinical presentation. In particular, patients with HCV chronic infection may develop over time either nonmalignant or malignant diseases. In particular, non-neoplastic pathological conditions include insulin resistance, Type 2 diabetes, myocarditis, cardiomyopathies, cardiovascular diseases (i.e., stroke, ischemic heart disease), chronic obstructive pulmonary disease, idiopathic pulmonary fibrosis, asthma, interstitial lung diseases, neurological and hematological disorders. Furthermore, the identification in clinical practice of subjects with occult HCV infection, both naive untreated individuals and patients with sustained virological response to direct-acting antiviral agents, contributes to making even more complex the understanding of the immunopathogenesis concerning this virus [139]. Hepatic (liver and cholangiocarcinoma cancers) and extrahepatic tumors (distinct subtypes of B-cell non-Hodgkin lymphomas, pancreatic cancer and probably breast-, thyroid-, ovarian- and renal-neoplasms) have been reported in patients with HCV-related previous infections [140–142]. Therefore, HCV causes systemic pathological diseases [143]. HIV-positive untreated patients undergo a state of immune dysregulation and immunodeficiency leading to the impairment of their IS function. These subjects also develop an increased risk of several nonmalignant or malignant diseases. Before the introduction of effective antiretroviral therapy, the most important pathological burden associated with HIV was due to the emergence of opportunistic infections (OIs) [144]. These pathological conditions emerge with the progressive decrease of the host’s CD4 T lymphocytes serum level. An increased risk of OIs is observed not only in HIV-positive patients with a count of these immune cells below 200/mm^3^, but also in individuals with more elevated levels of CD4 T lymphocytes (about 500/mm^3^) [145]. The decrease in the number of these immune cells below a critical count causes a global deficit of the IS. The OIs are generally characterized by an aggressive clinical course, resistance to treatment and an elevated rate of relapse. Protozoal- (*Pneumocystis carinii pneumonia*, Cryptosporidium, *Toxoplasma gondii*, *Isospora belli*), fungal- (*Candida*, *Cryptococcus*), bacterial- (*Mycobacterium tuberculosis*, *Mycobacterium avium*-intracellulare, *Salmonella*), viral- (herpes simplex virus, herpes zoster, cytomegalovirus) are the most common pathogens associated with HIV infection. However, even after the advent of effective antiviral treatment (ART), OIs are still observed, mainly in subjects who have not yet been diagnosed with HIV and in individuals not receiving therapy [144]. The introduction of ART has increased the life expectancy of HIV-infected patients, but they present a higher risk to develop several pathological conditions, including: autoimmune diseases involving lungs (sarcoidosis), thyroid gland (Graves’ disease), liver (autoimmune hepatitis), connective tissue (systemic lupus erythematosus, rheumatoid arthritis, polyarteritis nodosa and other types of vasculitis, antiphospholipid syndrome) or hematopoietic system (autoimmune cytopenias) [146]; neurological diseases, such as cognitive impairment, cerebrovascular disease and peripheral neuropathy [147]; cardiovascular diseases (heart failure, stroke and arrhythmias) [148]; insulin resistance, as well as metabolic derangement and the occurrence of Type 2 diabetes associated with ART [149]; renal injury through direct HIV-related cytotoxicity or immune complex-mediated glomerulonephritis, as well as nephrotoxicity and co-morbidities associated with ART for a long-last period [150]. HIV infection also correlates with an increased risk of developing some neoplasms. The term ‘HIV-associated cancers’ indicates a wide spectrum of malignancies occurring in these subjects, such as non-Hodgkin lymphomas, Kaposi’s sarcoma, invasive cervical cancer (known as AIDS-defining cancers) and lung-, anal-, vulvar-, penile-, hepatocellular cancers, Hodgkin’s lymphoma, oropharyngeal cancer and squamous-cell skin cancer, Merkel-cell carcinoma, the myelodysplastic syndrome, polycythemia vera and (mainly in sub-Saharan Africa) squamous-cell carcinoma of the conjunctiva (known as non-AIDS-defining cancers) [151,152]. To date, also viruses with DNA genomes, like HBV and some members of the herpesvirus family, including Epstein–Barr virus (EBV) and herpesvirus-8, are recognized as oncogenic agents. EBV infection is linked with an increased risk of non-Hodgkin’s and Hodgkin’s lymphomas, nasopharyngeal carcinoma and gastric cancer. A large spectrum of EBV latent genes is induced during the course of infection and it is mainly expressed during its later stages. This biological behavior allows the survival of the viral genome and prevents the protective activity of the host’s IR by a limited expression of the viral genome and of proteins. Interaction between cell oncogenes and EBV latent genes causes the perturbances of some steps of the host’s cell cycle, such as the transition from G1 to S phase and the inhibition of cell apoptosis [136,153]. In addiction, herpesvirus-8 has been associated with the development of two malignancies: Kaposi sarcoma, a plasmablastic form of multicentric Castleman disease, and primary effusion lymphoma. These tumors affect subjects with acquired immunodeficiencysyndrome, iatrogenic immunosuppression, including organ transplantation and elderly people [134]. Although it is still unknown whether the influenza virus, causing the Spanish flu pandemic, may have had a significant impact on the oncogenic risk in patients infected with this pathogen in the years after 1918, some studies are investigating this problem. It has been observed that the subjects who acquired this infection had a more elevated old-age mortality due to cancerous and noncancerous causes in comparison with individuals who did not come in contact with this virus. Patients with a low noncancerous mortality had a high cancer one and *vice versa* [154]. Further investigations are needed to better understand the clinical significance of the Spanish pandemic. Some studies have investigated the possible effect of SARS-CoV-2 infection in patients with malignancies and have reported that these individuals exhibit a three-times higher susceptibility to this pathogen and a potential worse prognosis in comparison with individuals without cancer. The more elevated MR in people with tumors may be due to several reasons. The systemic immunosuppression, induced by the tumor itself or by surgical procedures and by the chemotherapy, represents one of the main causes of the higher probability to get SARS-CoV-2 infection by these patients [155]. On the other hand, a potential impact of this pathogen on the risk of cancer development in humans has been also hypothesized, but this possibility has not been tested or demonstrated up to now, since this virus has been identified only recently [155,156]. This is why to date no adequate long-term follow-up exists concerning the subjects who have acquired SARS-CoV-2 infection and no studies, designed with this aim, are available. It is now well known that several and different viruses may induce a persistent inflammation in infected human hosts and this condition is associated with an increased risk of cancer onset [157]. The process of carcinogenesis mediated by viruses is generally multifactorial, it takes a rather long time to occur and shares some pathogenetic mechanisms. On the basis of this assumption, it has been hypothesized that SARS-CoV-2 also may act as an oncogenic pathogen, such as HCV, HIV, EBV and HBV. All these pathogens produce proteins able to induce proinflammatory processes by damaging the functions of several microorganelles, such as cytoskeleton, mitochondria, Golgi’s apparatus, Endoplasmic reticulum, as well as chromatin conformation, disposition and activities both in the cytoplasm and in the nucleus of the host’s cells. These viruses may also induce the recruitment of some immune cells and the activation of signaling pathways, promoting the generation of an persistent inflammatory process [155,156]. Similar mechanisms may be associated with a long-lasting inflammation of human tissues, mediated by SARS-CoV-2 and they may be involved in the generation of human malignancies. Some researchers have suggested that this pathogen may stimulate the function of the renin-angiotensin system. It has been hypothesized that the hyperactivation of this system may exert a facilitating role in the process of human carcinogenesis [157]. In particular, during the course of inflammation SARS-CoV-2 infection can induce a permissive tissue microenvironment, where malignant cells can undergo a long-lasting process of transformation, acquiring progressive genotypic and phenotypic modification and increasing their ability to proliferate. Furthermore, it has also been suggested that a similar process might be active in patients with previously or currently diagnosed cancers. In these individuals, some dormant cancer cells (DCCs) can survive the treatment of primary tumors and in premetastatic niches. The inflammatory process, due to SARS-CoV-2 infection, may awaken DCCs in tissue microenvironment by stimulating some crucial cells, such as neutrophils and monocytes/macrophages. These cells release some products, such as extracellular neutrophil traps and proinflammatory cytokines, and promote the reactivation of these DCCs [158]. Taken together, all these events may contribute to explain the higher susceptibility of patients with cancers to SARS-CoV-2 and the poorer prognosis in comparison with individuals without malignancies.

## Considerations on the SARS-CoV-2 redetection in patients with previous contact with this pathogen

It is necessary to consider the affected patients, with confirmed at laboratory tests, who remain or become again RT-PCR positive for viral RNA search at NSWAB even after a prolonged period of time or after their clinical recovery [159,160]. The clinical significance and the reason of this observation remain poorly known [161,162]. The interest in clarifying this topic is progressively increasing worldwide and some studies are investigating the possible causes of this event [163]. SARS-CoV-2 reappearance may depend on the reinfection (defined as a new infection by the etiological agent of COVID-19 after its clearance following the previous contact with this pathogen) or on the recurrence (defined as reactivation of SARS-CoV-2, after the apparent control of the virus by the host’s antiviral IR). Cases of reinfection by phylogenetically distinct clades of this pathogen have been proved worldwide taking advantage of the entire viral genome sequencing [164–166]. Wide variations in viral loads, clinical manifestations and periods of the time elapsing between the infectious episodes have been described in these patients. These individuals were either asymptomatic or experienced mild/moderate symptoms [165,166]. However, other studies have suggested that some subjects, who had apparently recovered from the SARS-CoV-2-related infection and have become positive again at a successive control using RT-PCR test at NSWAB even several days after the first negativization, may have experienced a reactivation of this pathogen, rather than a reinfection [167]. Most of these studies have included a limited number of patients with a short-lasting period of follow-up [168–170]. However, the number of reports describing subjects with possible SARS-CoV-2 redetection is progressively increasing. A recent large cohort study enrolled 1146 infected patients, out of these 125 presented a new positive NSWAB after two negative tests after their clinical recovery and during the course of follow-up. It may be hypothesized that these patients have experienced a reactivation of SARS-CoV-2, although it is not possible to exclude a reinfection [159]. Similar results have been obtained in further small-sized studies as well as in a Chinese retrospective research, including 758 individuals with SARS-CoV-2 infection [170,171]. Fifty-nine of them presented recurrent positive RT-PCR after their clinical recovery [172]. According to available data, resulting from about 80 studies, Gidari *et al.* have reported that from January to September 2020, 1350 patients, who have recovered from the COVID-19, presented again a positive RT-PCR at NSWAB worldwide. New positive respiratory tests were observed in mean of 34.5 days after the second negative sample had been observed. However, the data were available only for 123 subjects [173]. Similar results have been reported in a further systematic review by Dao *et al*. Overall, the percentage of repositive subjects among discharged individuals with SARS-CoV-2 infection ranged from 2.4 to 69.2% in the considered studies, but in most of them was equal to 10–11%. The time elapsing from patients’ discharge to the first redetection test varied between 1 and 35 days [174]. The reactivation of SARS-CoV-2 replication has been recently described in one patient suffering from meningoencephalitis with unexpected severe clinical manifestations, even after the apparent resolution of the infection, as well as in one subject who has undergone immunosuppression, following the treatment for B-cell acute lymphoblastic leukemia [175,176]. It may be hypothesized that both cases are associated with a reactivation of SARS-CoV-2, persisting in a latent status in the previously infected host rather than a reinfection. Based on this consideration and taking into the due account the biological behavior of other viruses, such as HIV and HCV, we have to consider that SARS-CoV-2 might persist at very low or undetectable titers with the common detection tests in the so-called sanctuary organs and periodically reactivate and resume its replication. The relevance of this issue may have a double effect: the reactivation of the viral replication in different anatomic sites in some individuals, with the generation of significant viral loads in these carriers; these individuals may remain asymptomatic or become symptomatic and they might theoretically contribute to spreading the virus; the lack of the complete eradication of this virus and its persistence at undetectable titers in the host may correlate with the re-emergence of its replication and cause unexpected severe clinical manifestations. These events may occur: in a relatively short-lasting time, as it has already shown in previous case reports describing immunocompromised patients, as well as in subjects with persistent severe clinical manifestations after the resolution of SARS-CoV-2 infection. Some pneumological-, neurological- (cerebrovascular accident such as acute hemorrhagic-necrotizing encephalopathy, Guillain–Barré syndrome, acute transverse myelitis, epilepsy and acute encephalitis and cardiovascular (acute coronary syndrome, myocarditis, stress-cardiomyopathy, arrhythmias, heart failure and venous thromboembolism) complications have been described to occur in patients with COVID-19 [175–181]. The real impact of these clinical manifestations on public health will have to be carefully assessed in the next future and in the long term with well-designed and well-sized studies; in a long-lasting time, with an increased risk of progressively developing adverse pneumological, cardiovascular and neurological outcomes or malignancies, as observed for other oncogenic viruses that cause latent infections (HCV, HIV) in carriers. This possibility should be considered in the next years or decades [182–184].

Overall, all these considerations underline the importance of understanding whether the patients with SARS-CoV-2 infections may experience both reinfection and reactivation, as the clarification of this topic may have a strong impact on clinical practice. The identification of individuals at high risk of SARS-CoV-2 recurrence/reactivation may represent a fundamental measure for the control of the pandemic spread of this pathogen.

## Considerations on the possible involvement of climatic factors in increasing the risk of SARS-CoV-2 spreading

Climatic factors have been reported to strongly influence the transmission of several IDs and to have an impact on their geographical and seasonal distributions, mainly in winter in temperate regions and in long-lasting trends [185]. Available studies have underlined the effects of the environmental factors, such as temperature and humidity, in influencing innate and adaptive IRs of the host to viral infections involving the respiratory tract [186]. High levels of these two physical parameters have been reported to promote the transmission of the SARS-CoV-2 with discordant results [187]. Some studies suggest that high temperatures can prevent the diffusion of this pathogen in different geographical areas, with a negative association between the average temperature and the number of cases of SARS-CoV-2 infections [188,189]. An higher stability of this virus was observed when the specimens were maintained at low-temperature and low-humidity conditions [190]. A hotter temperature and a more elevated humidity decreased its half-life (when the temperature ranged from 4 to 27°C) [183,184]. Mean low temperatures ranging from 5 to 11°C, low specific humidity (3–6 g/kg) and low absolute humidity (4–7 g/m^3^) correlate with a more elevated SARS-CoV-2 ability to spread in different geographical areas worldwide [191,192]. *In vivo* and *in vitro* data about several coronavirus types have reported that the increase of temperature reduces the virus' viability on different materials (including SARS-Cov-2) [190,193]. Other studies have examined the overall effects of several factors, including temperature, humidity and rainfall, but the results of these studies are still controversial and no definitive conclusions have been reached about the transmission of respiratory viruses when these environmental conditions are considered all together [194]. In particular, air relative humidity (RH) has resulted as a physical parameter of great interest in some studies, as high values of this factor are associated with inhibitory effects for SARS-CoV-2, MERS-CoV and IVA. Nevertheless, the impact of RH in influencing SARS-CoV-2 activity is less pronounced in comparison with the parameter represented by temperature, although no definitive results have been obtained. What is more, both high (>85%) and low (<60%) RH determine a significant decrease of infectivity in a model surrogate for IV and SARS-coronavirus [195]. Low absolute humidity (4–7 g/m^3^ humid air) and specific humidity (3–6 g/kg dry air) turn out to characterize the areas in the world with a high spread of SARS-Cov-2, whereas the RH levels of the same impacted areas are not discriminating against poorly affected territories across the northern hemisphere [192]. A different result arises from selected cities in Brazil [187], where a higher RH seems to promote the COVID-19 diffusion; on the contrary, a tendency for a decrease of cases with the increasing RH is described in New South Wales during the falling phase of the epidemic [196]. Such contradictory shreds of evidence could convince that the water vapor is a weak forcing factor for SARS-CoV-2 transmission not to be theoretically neglected, as the European Commission conceives the absolute humidity among the few possibly significant environmental claims in this context. The sun UV radiation represents a more definite and characterized environmental variable on the virus survival. Only the sun radiation in the range of the UV-A (400–315 nm) and UV-B (315–280 nm) reaches the surface of the earth, whereas the UV-C component (<280 nm) is absorbed by the atmosphere. The UV-C radiation exerts antiviral and antibacterial activities and it is commonly used as a germicidal tool against viral aerosols with very promising results; however, UV-C cannot be considered as an outdoor environmental factor [197,198]. Nevertheless, the effectiveness of solar radiation in reducing the viability of the IVs under laboratory conditions is known [199]. Also for SARS-CoV-2 laboratory data on anhydrous surfaces and culture media show similar results and significant differences in the virus activity comparing the sunlight conditions during the winter, the middle season and the summer at mid latitudes [200]. These laboratory results agree with theoretical computations and with ecological studies, which suggest a correlation between lower COVID-19-infection rates with higher solar radiation [201,202]. A wide population study has considered approximately 18,000 cases of COVID-19 in China [203] with apparently contradictory results, deriving from the ecological approach: the solar radiation is still considered to exert an effective contrast against the virus survival [199], sunshine days induce higher sociality and a consequent higher probability of its transmission. The efficacy of the UV solar radiation has been considered to be also therapeutic after its use had been successfully observed in the 1930s and 1940s for a variety of diseases. Biological adverse or beneficial effects of the UV-B radiation have been reported by several studies. In brief, UV-B radiation may either induce the well-known damage of human skin or serve as a possible activating factor for the dormant HIV-1 genome, at appropriate doses [204–206]. However, UV-B rays are involved in the activation of vitamin D (VD) in the human skin. The overall effect of exposure to sunlight seems very beneficial against the IVs, as well as against other pathogens. This effect has been correlated with the increased amounts of activated forms of VD induced by UV-B rays [207]. Exposure to sun radiation has been associated with a significant increase of the total VD concentration in a cohort of Norwegian women and with a reduced risk of IVs infection [208]. Even in HCV patients, the hours of exposure to sunlight have been demonstrated to correlate with a better response to a therapy based on the administration of peginterferon/ribavirin and boceprevir/telaprevir in a cohort of patients with HCV chronic hepatitis who were treated in the summer, in comparison with a group of HCV-positive subjects with persistent liver disease with similar baseline characteristics treated in winter months [209]. The production of VD stimulated by sun radiation seems very effective in reducing viral infectivity and in preventing the development of more severe forms of viral-related infections. Therefore, its use has been proposed several months ago with a therapeutic or preventive role in patients at a higher risk of severe clinical manifestations of SARS-CoV-2-related infection, such as aged people or for the treatment of these individual [17,22,210–212]. Whether solar radiation can be effective for viruses inactivation and for VD production, air pollution could severely prevent such beneficial effects, because airborne particles shield solar radiation up to significant levels [213]. Moreover, the particulate matter represents an additional risk factor, because it is associated with cardiovascular and respiratory diseases [214]. These pathological conditions can increase the susceptibility to respiratory virus infections [215]. The effect of air pollution in increasing the lethality of COVID-19 has been suggested for highly polluted areas in Italy and similar results have been obtained for China and for the USA both for SARS-CoV-1 and SARS-CoV-2 [216,217]. An additional point is whether air particulate matter can transport viruses. Genetic material from the SARS-CoV-2 virus detected on PM10 samples, which have been obtained from the city of Bergamo (Italy). Bergamo has been severely hit by SARS-CoV-2 infection [218]. Further data, collected from other Italian areas during the COVID-19 pandemic [219], show that in crowded sites SARS-CoV-2 is detectable in the PM10, whereas the virus is absent in the particulate in nonpolluted regions. A very recent nationwide observational study, carried out in Italy, has shown that a significant positive association exists between COVID-19 incidence rates and levels of PM2.5 and NO_2_ taking into account the period 2016–2020 and the months of the epidemic [220]. Nowadays, a definitive conclusion concerning this important problem is not yet possible, but as it stands the association of viruses on colloidal and particulate materials is well known in aqueous medium and we know it can enhance the transport of viruses to long distances [221]. Therefore, the identification of particulate composition can be a critical factor in the control of infectivity, mainly for airborne particles, because despite viruses can be light-shielded during UV irradiation, they can be inactivated by Fenton-like processes, triggered by iron atoms at the particulate surface [222]. These very interesting observations need further well-designed studies to definitively clarify all these points [222].

## Conclusion & future perspective

The lack of definitive and effective results for the control of SARS-CoV-2 infection depends on several reasons, such as: its recent identification, the restricted period of follow-up in both symptomatic- and asymptomatic-infected subjects and the heterogeneity of the available studies (design, inclusion criteria, diagnostic tools, period of follow-up and end points). Using insights and lessons from HIV, HCV (bloodbornes), IVs (airbornes) characteristics, and from prior pandemics, SARS-CoV-2 has been deeply investigated to underline similarities and differences and to hypothesize a pattern of behavior for this virus. Several studies have investigated the quasispecies of the above pathogens [223–226]. RNA viruses possess the capacity to exchange genetic material with each another, continuously generating new viral variants by recombination and by reassortment. HIV, HCV and IVs, SARS-CoV-2 and the other coronaviruses (CoVs) exhibit lengther genomes (about 30,000 bases) [7]. This viral characteristic is linked to a lower mutation rate among the members of the Coronaviridae family, including SARS-CoV-2, in comparison with other small RNA viruses, although it exhibits an elevated genome heterogeneity. This peculiar feature has been associated with the capacity of CoVs to produce a proofreading exoribonuclease (ExoN), known as nsp14, mediating a high fidelity in RNA genome replication [227]. The increase in the mutation frequency in patients suffering from infections associated with these pathogens may induce a double effect: either forcing the viruses beyond a tolerable limit and results into the ‘error catastrophe’, with the consequent reduction in its fitness, its impossibility to replicate and its extinction; or promoting the emergences of variants escaping from the pressure due to the IS to specific pharmacological treatments. This aspect has to be taken into account in the research and the development of drugs potentially active against SARS-CoV-2. A wide spectrum of existing pharmaceutical compounds has been repurposed for the treatments of the infection associated with this pathogen. These drugs have different targets, such as: direct activities against some viral proteins of SARS-CoV-2, the capability to inhibit viral entry or release and the capability to modulate the function of the IS and to decrease the inflammatory response, and are briefly described in this section [228]. Among the pharmaceutical compounds in group A, several nucleoside analogs have been introduced in clinical practice as treatment of patients with some RNA-related viral infections, such as ribavirin and remdesivir [46,229]. This last, an adenosine nucleotide analog, competes with ATP for binding to RNAp and for being incorporated into the nascent viral genome, causing its termination after the additional insertion of up to three further nucleotides into the RNA strand [229]. A steric hindrance prevents the RNAp to continue its activity and may block the proofreading action of the nsp14 component and inhibit the excision of the analog from the viral chain. Nevertheless, a residual exonuclease function of nsp14 probably persists. Studies *in vitro* have shown that the absence of proofreading activity in mutant viral strains is associated with a more elevated remdesevir-associated inhibition of replication in comparison with wild-type ones [230]. The use of remdesivir in clinical practice has demonstrated beneficial effects in the course of SARS-CoV-2 infection, as it is associated with the improvement in the 28-day recovery rate and with the decreased need for oxygen support, although no definitive results are available to date. Further drugs with a possible activity against this pathogen include Lopinavir. It acts as an inhibitor of a crucial viral enzyme with protease functions, catalyzing the processing of some SARS-CoV-2 proteins [231]. Therefore, it interferes with viral replication. Lopinavir is coadministered with Ritonavir to increase its plasma half-life and to obtain concentrations effective for the inhibition of viral replication. Some preliminary data suggest potential efficacy of Lopinavir in the treatment of patients with COVID-19, but definitive results are still lacking [232]. Based on of some in ‘*in vitro*’ reports assessing the possible antiviral activities of IFN-β-1α, this molecule has been used in clinical practice in association with hydroxychloroquine and lopinavir/Ritonavir [233]. However, the small number of patients enrolled in the available trials and the discordant results on the efficacy of this treatment against SARS-CoV-2 make these studies inconclusive [234]. Some drugs exhibit the possible role in inhibiting SARS-CoV-2 viral entry into the cells or its release from them (group B). They include some compounds, such as nelfinavir mesylate, that display the capability to inhibit the transfer and the spread of this pathogen from cell to cell or Oseltamivir, which is active against NA of influenza A and B virus and that has been tested in small studies also in subjects with COVID-19. However, to date, no conclusive results concerning the effects of these compounds are available [232]. The drugs with the capability to modulate IS activities, by decreasing the inflammatory response, are represented by: colchicine, hydroxychloroquine, tocilizumab, corticosteroids and VD. Colchicine interferes with a large number of inflammatory events in the cells, such as inflammasome activation, cytokine/interleukins production, inflammatory cell chemotaxis and phagocytosis. Some preliminary studies have shown that colchicine may exert beneficial effects in subjects with COVID-19, by counteracting inflammatory events, through NLRP3 the inhibition of inflammasome and cytokine storm [235]. Hydroxychloroquine decreases the production and release of proinflammatory cytokines, promoting the suppression of an excessive host immune reaction. Some preliminary beneficial effects of this drug in the treatment of COVID-19 have not been confirmed by a meta-analysis. This study has shown that the therapy with hydroxychloroquine results in increased mortality in patients with SARS-CoV-2 infection [236]. Tocilizumab is a monoclonal antibody active against the IL6 receptor. After the onset of the SARS-CoV-2 pandemic, the use of this drug has been proposed in individuals with severe forms of COVID-19 in a compassionate manner [237]. Some studies have shown potential beneficial effects of this drug in improving the outcome and MR in patients with COVID-19 [238,239], but further randomized clinical trials have not confirmed these results [240]. Potential benefits of corticosteroid use have been reported in an increasing number of trials. A recent study has assessed the use of dexamethasone in patients with COVID-19. This drug has decreased the MRs in intubated patients in comparison with ones, undergoing standard treatments. However, the administration of dexamethasone has resulted in significant clinical benefits only in SARS-CoV-2-positive subjects with respiratory insufficiency. On the other hand, its use in patients with COVID-19, but without the need for respiratory support, has not been associated with a better outcome in comparison with standard therapies [241]. A systematic review and meta-analysis have assessed the effectiveness and safety of corticosteroids in individuals with SARS-CoV-2, including 44 studies with 20,197 subjects. This study has shown beneficial effects with the use of corticosteroids on short-term mortality and a reduced need for mechanical ventilation in patients with COVID-19. However, the administration of these drugs has been associated with a slightly higher risk of infections and more elevated use of antibiotics [242]. Two recent systematic review and meta-analysis have shown both that patients with VD deficiency were associated with increased SARS-CoV-2 infection risk, higher disease severity and mortality than subjects with COVID-19, but with normal serum levels of this fat-soluble micronutrient and that its administration is able to reduce the death rate, the severity of the disease [243], and serum levels of inflammatory markers in patients with COVID-19 [244]. On the other hand, further statistical analysis has reported only a trend for an association between low serum 25(OH)D levels and COVID-19-related poor health outcomes [245]. A careful examination of all these data from the above-mentioned studies should help in the searching for antiviral drugs effective against SARS-CoV-2, in overcoming the problems associated with the resistance to these compounds as well as in modulating and in attenuating the inflammatory IR, during the onset and development of COVID-19. The emerged data, describing the history and the management of these viruses, offer a broad perspective for discussion. SARS-Cov-2 is a recently identified virus and its structure and its biological behavior are not fully understood yet and this is why some open questions remain. The present paper should provide a useful synthesis of the body of data and of shreds of evidence concerning useful lessons to counteract the pandemic caused by SARS-CoV-2 by planning effective preventive and therapeutic strategies. This approach should finally allow us to precede this virus, preventing its hamper effects and not chasing it.

**Executive summary**

**HIV, HCV, Influenza viruses & SARS-CoV-2 show differences in their transmission**

- SARS-CoV-2 and Influenza viruses (IVs) are airborne pathogens.
  HIV and HCV are bloodborne infectious agents.

**HIV, HCV & IVs are RNA viruses characterized by common elements**

- A small-size genome of about 10,000–15,000 bases and an elevated mutation rate (around 10–3 and 10–5 errors per nucleotide and replication cycle) due to the small length of their RNA leading to compression and overlap of genes in their genome.
- Error-prone genomic replication as their RNAps lacks the proofreading activity or postreplicative RNA repair mechanisms.
- Short replication time and large progeny.

**SARS-CoV-2 genome & mutations**

- A longer genome (about 30,000 bases).
- A replicative machinery consists of a catalytic subunit (nonstructural protein 12 [nsp12]), exerting the RNAp activity.
- Nsp12 also is error-prone during viral genome replication, however, the high rate of mutations detectable in the course of this process is attenuated by the existence of an additional subunit, known as nsp14.
- A proofreading exoribonuclease (ExoN) activity with error-correcting capability, this structure mediates a higher fidelity in RNA genome replication.
- The poor fidelity of viral RNAps in mediating this process is considered as the primary contributing factor for the variability in the genomes of RNA viruses. SARS-CoV-2 also is characterized by a wide variability due to: the existence of several genotypes and subtypes; the elevated daily mutation rates (missense, synonymous and noncoding mutations), leading to the generation of different but closely related variants, known as quasispecies; and the capacity to exchange genetic material among viral genomes, during viral replication. This event is defined gene recombination and it is characterized by the mixing of gene segments, belonging to two different parental genomes, producing a viral progeny with new combinations of genetic material.

**Climatic factors**

- Sun UV radiation represents a variable on the virus survival.
- Studies suggest a correlation between lower COVID-19-infection rates and higher solar radiation.
- UV-B rays are involved in the activation of vitamin D in the human skin. The overall effect of exposure to sunlight seems very beneficial against the influenza virus, as well as against other viruses. This effect has been correlated with the increased amounts of activated forms of vitamin D, induced by UV-B rays and several beneficial immunomodulatory and antiviral activities against SARS-CoV-2 have been proposed also for this compound.
- Low temperatures ranging from 5 to 11°C, low specific humidity (3–6 g/kg) and low absolute humidity (4–7 g/m^3^) correlate with a more elevated SARS-CoV-2 ability to spread in different geographical areas worldwide.
- Both high (>85%) and low (<60%) relative humidity determines a significant decrease of infectivity in a model surrogate for influenza virus and SARS coronavirus. The increase of temperature causes the reduction of virus viability on different materials (including SARS-Cov-2).
- The particulate matter (PM) is also an additional risk factor for SARS-CoV-2 infection, in fact, a significant positive association exists between COVID-19 incidence rates and levels of PM2.5 and PM10.

**Historical epidemics & waves**

- Since 1889, several IVs pandemics have been described worldwide.
- All these pandemics have lasted at least 2 years.
  A number of waves, ranging from 2 and 4 during their course, were described, generally with an higher mortality rate during the second and the third respect the first one.

**SARS-CoV-2 persistence in humans**

- Redetection of SARS-CoV-2 is observed with increasing frequency in patients who have been thought to have cleared this pathogen, but the clinical significance of this observation, as well as the reasons of this event, remain uncertain and poorly known.
- Some studies have reported cases of individuals with well-documented SARS-CoV-2 reinfection, no data are available concerning the possibility of its reactivation, as observed for HIV and HCV. Further studies are requested to clarify this point.
